# Using a System Pharmacology Method to Search for the Potential Targets and Pathways of Yinqiaosan against COVID-19

**DOI:** 10.1155/2022/9248674

**Published:** 2022-03-15

**Authors:** Li-hua Cao, Xing-yuan Jia, Hong-juan He, Zhen-zhen Wang, Yi-ying Zhao, Xue Yang, Xiu-min Li, Ming-san Miao

**Affiliations:** ^1^Academy of Chinese Medical Sciences, Henan University of Chinese Medicine, Zhengzhou 450046, Henan Province, China; ^2^Department of Pharmacy, Henan Province Hospital of Traditional Chinese Medicine, Zhengzhou 450046, Henan Province, China; ^3^School of Pharmacy, Henan University of Chinese Medicine, Zhengzhou 450046, Henan Province, China; ^4^Microbiology and Immunology Department, New York Medical College, Valhalla, NY 10595, USA

## Abstract

The first reported case of coronavirus disease 2019 (COVID-19) occurred in Wuhan, Hubei, China. Thereafter, it spread through China and worldwide in only a few months, reaching a pandemic level. It can cause severe respiratory illnesses such as pneumonia and lung failure. Since the onset of the disease, the rapid response and intervention of traditional Chinese medicine (TCM) have played a significant role in the effective control of the epidemic. Yinqiaosan (YQS) was used to treat COVID-19 pneumonia, with good curative effects. However, a systematic overview of its active compounds and the therapeutic mechanisms underlying its action has yet to be performed. The purpose of the current study is to explore the compounds and mechanism of YQS in treating COVID-19 pneumonia using system pharmacology. A system pharmacology method involving drug-likeness assessment, oral bioavailability forecasting, virtual docking, and network analysis was applied to estimate the active compounds, hub targets, and key pathways of YQS in the treatment of COVID-19 pneumonia. With this method, 117 active compounds were successfully identified in YQS, and 77 potential targets were obtained from the targets of 95 compounds and COVID-19 pneumonia. The results show that YQS may act in treating COVID-19 pneumonia and its complications (atherosclerosis and nephropathy) through Kaposi sarcoma-related herpesvirus infection and the AGE-RAGE signaling pathway in diabetic complications and pathways in cancer. We distinguished the hub molecular targets within pathways such as TNF, GAPDH, MAPK3, MAPK1, EGFR, CASP3, MAPK8, mTOR, IL-2, and MAPK14. Five of the more highly active compounds (acacetin, kaempferol, luteolin, naringenin, and quercetin) have anti-inflammatory and antioxidative properties. In summary, by introducing a systematic network pharmacology method, our research perfectly forecasts the active compounds, potential targets, and key pathways of YQS applied to COVID-19 and helps to comprehensively clarify its mechanism of action.

## 1. Introduction

Coronavirus infectious disease 2019 (COVID-19) is caused by 2019-nCoV virus and it seriously endangers human health. On January 12, 2020, the World Health Organization (WHO) confirmed and named its pathogen 2019-nCoV [1], which belongs to the novel coronavirus pneumonia of genus *β*. On January 31, 2020, it was designated a Public Health Emergency of International Concern (PHEIC). According to the latest statistics of the World Health Organization, as of October 28, 2021, there were approximately 244385444 confirmed cases and more than 4961489 deaths worldwide, including over 7053806 confirmed cases and over 108334 deaths in the United States.

The main clinical features of these cases are a high body temperature, cough without mucous, breathing difficulties (dyspnea), headache, and pneumonia. Disease attack may lead to progressive respiratory failure due to alveolar damage (which has been observed by transverse chest computerized tomography images) and cytokine storm syndrome and it could also develop a severe form with sometimes fatal outcomes [2, 3]. The cause of the disease was identified by clinicians as virus-induced pneumonia in line with the clinical signs and other standards, including increased body temperature, decreased lymphocytes and white blood cells (although the levels of the latter seemed normal sometimes), new pulmonary infiltrates on chest radiography, and no evident improvement after treatment with antibiotics for three days [4].

In traditional Chinese medicine (TCM), COVID-19 falls under the category of “pestilences,” which occur because of direct or indirect contact with epidemic pathogens. Since the onset of the disease, the rapid response and intervention of TCM have played a vital role in effectively controlling the epidemic, and the classic prescription has become one of the highlights in preventing and controlling the epidemic [5, 6]. Yinqiaosan (YQS) is a traditional prescription for treating the influenza virus from the “Wen Bing Tiao Bian.” YQS is a herbal formula comprised of 10 medicinal herbs (Jinyinhua (JYH, *Lonicera japonica* Thunb.), Lianqiao (LQ, *Forsythia suspensa* (Thunb.)), Jiegeng (JG, *Platycodon grandiflorum* (Jacq.) A. DC), Bohe (BH, *Mentha haplocalyx* Briq.), Dantouchi (DDC, *Glycine* max (L.) Merr.), Danzhuye (DZY, *Lophatherum gracile* Brongn.), Niubangzi (NBZ, *Arctium lappa* L.), Jingjie (JJ, *Schizonepeta tenuifolia* Eriq.), Lugen (LG, *Phragmites communis* Trin.), and Gancao (GC, *Glycyrrhiza uralensis* Fisch., *Glycyrrhiza inflata* Bat., or *Glycyrrhiza glabra* L.). The National Health Commission of the People's Republic of China and the National Administration of TCM issued the “Diagnosis and Treatment Program for COVID-19 (Trial Version 3).” They began to recommend TCM therapy, among which the recommended prescriptions are Maxing Shigan decoction and YQS.

Tang et al. [7], based on the TCMATCOV platform, analyzed the potential effects of commonly used traditional formulations for treating COVID-19 and discovered that YQS is a frequently used therapy for pneumonia. For the past few years, systems pharmacology [8, 9] means have been widely introduced in the prescription and mechanism of TCM. System pharmacology is an emerging systematic methodology and is guided by the “multicomponent multitarget network” theory, which conforms to the integrity and systematicity of TCM prescriptions. In addition, it combines oral bioavailability screening and multiple drug target prediction, providing an overall method for exploring the targets and potential mechanisms of TCM, and it can be used as an alternative strategy to analyze the therapeutic effects of the active ingredients [10, 11]. Wang et al. [12] discussed the targets and signaling pathways of Maxing Shigan decoction for treating pneumonia through systems pharmacology and found that the possible targets were IL-6, TNF, MAPK8, etc. Xia et al. found that Akt1 was a potential target of Lianhua Qingwen to treat and prevent COVID-19 using network pharmacology and molecular docking analyses [13].

Aiming to explore the possibility and mechanism of YQS in treating COVID-19, this paper uses systems pharmacology techniques and approaches to forecast the possible targets and signaling pathways of YQS and provides data for subsequent experimental research.

## 2. Materials and Methods

### 2.1. Finding Potential Targets of YQS

Taking the herbs (Jinyinhua, Lianqiao, Jiegeng, Bohe, Dantouchi, Danzhuye, Niubangzi, Jingjie, Lugen, and Gancao) in YQS as keywords, the TCMSP database (http://tcmspw.com/tcmsp.php) was used to retrieve their relevant chemical compositions, and oral bioavailability (OB) ≥ 30%, drug-likeness (DL) ≥ 0.18, and drug half-life (HL) ≥ 4 h were adopted as screening conditions to obtain active ingredients that meet the conditions [14–16]. Among them, the active ingredients of Danzhuye were obtained through a combination of a literature search [17] and the TCMSP database.

Through the TCMSP database and the PubChem database (https://pubchem.ncbi.nlm.nih.gov/) combined with the Swiss Target Prediction database, the targets of the active ingredients were obtained. UniProt KB (http://www.UniProt.org/), which prevents, for example, overannotation of semblable proteins such as paralogs and putative products of pseudogenes, was utilized to normalize gene names and organisms. Through the UniProt database (https://www.UniProt.org/), the species was set as “*Homo sapiens* (Human)” to standardize the obtained drug targets.

### 2.2. Screening of COVID-19-Associated Genes

Through the GeneCards database (http://www.genecards.org/) and OMIM (https://www.omim.org/) database, using “novel coronavirus pneumonia” as the keyword, COVID-19-related disease targets were retrieved and obtained. Only “*Homo sapiens*” proteins linked to COVID-19 were selected. The UniProt database was applied to standardize the obtained disease targets in *Homo sapiens*.

### 2.3. Screening of Candidate Targets

The acting targets of the active ingredients of YQS and the disease targets of COVID-19 were processed through Venny2.1 software (http://bioinfogp.cnb.csic.es/tools/venny/index.html) to acquire their common targets, which were taken as alternative targets of YQS for treating COVID-19, and its Venny map was also acquired.

### 2.4. Protein-Protein Interaction (PPI)

The retrieved YQS active ingredient targets were linked to COVID-19 targets by the STRING (https://string-db.org/, ver.11.0) database [18]. The requirement was restrained to “*Homo sapiens*.” In this paper, a high confidence score with a correlation degree ≥0.400 [19] as the cutoff value was set to obtain the network.

### 2.5. GO and KEGG Pathway Enrichment Analysis

Gene Ontology (GO) enrichment analysis was conducted using the OmicShare tools (http://www.omicshare.com/tools). The KOBAS 3.0 database was applied to analyze the KEGG pathway enrichment of the candidate targets, and the RStudio 3.6.3 and ggplot2 packages were applied to illustrate the results.

### 2.6. Networks Construction and Analysis

Cytoscape 3.5.1, an open-source software platform to visualize complex networks, was employed to illustrate the networks. With this software, the network building was handled as follows: (1) compound-COVID19 PPI network, (2) compound-targets network, and (3) YQS (Yinqiaosan)-compound-targets-pathway network. In addition, four topological features of the hub network, such as “Degree” and “Betweenness Centrality,” were determined to choose YQS alternative targets with topological significance [20, 21]. To determine the relationship between network clusters and to identify high-connectivity hub genes, “Cytohubba” (a plug-in of Cytoscape) was introduced to check the node element. The top 10 nodes were ranked by EPC [22].

### 2.7. Molecular Docking

For the sake of validation of the drug-targeted correlations, the molecular docking simulation further proceeded on every drug docking with their targets. Each drug molecule, such as luteolin, naringenin, acacetin, kaempferol, and quercetin, was downloaded from the TCMSP database and converted to a PDBQT file by Chimera (version 1.10.2).

The structures of the proteins in each alternative target were downloaded from the RCBS Protein Data Bank (http://www.rcsb.org/pdb), and every protein file was opened with ADT (version 1.5.6), a free GUI for AutoDock. In every file, the water molecules were canceled, the polar hydrogen atoms were increased, and then they were written to a PDBQT file.

The intersection of possible alternative targets and drug targets of PCOS was chosen for in-depth investigations. To validate the interaction and observe the docking sites between drugs and targets, molecular docking was performed by an open-source program called Autodock vina 1.1.2. The docking results were observed with ADT.

**Figure 1:**
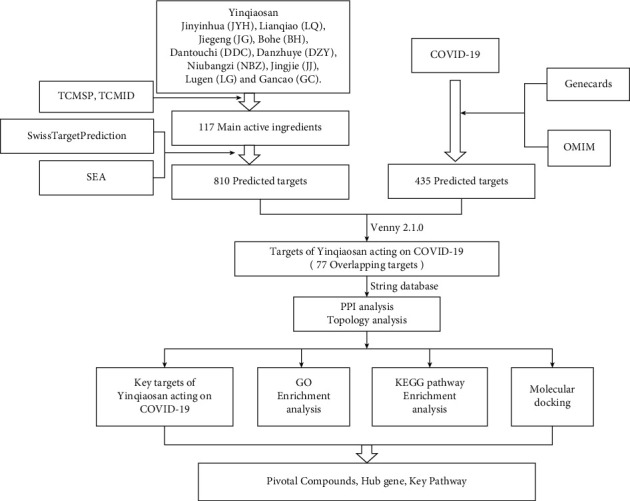
Process overview. To understand the active ingredients of each single drug in Yinqiaosan (YQS) through the TCMSP (Traditional Chinese Medicine Systems Pharmacology Database and Analysis Platform) and TCMID (Traditional Chinese Medicine Integrated Database), the targets of the active ingredients were identified by Swiss TargetPrediction and SEA (similarity ensemble approach). Similarly, COVID-19 targets were obtained through the GeneCards and OMIM (Online Mendelian Inheritance in Man) databases. Intersecting targets were then assessed by molecular docking and GO and KEGG enrichment analysis. GO (Gene Ontology) enrichment analysis was carried out with the OmicShare tools. The KOBAS 3.0 database was used for KEGG enrichment analysis, and RStudio was used to visualize the results. Molecular docking was conducted by an open-source program named Autodock vina 1.1.2. Networks were constructed to provide a visual view by Cytoscape 3.5.1 software.

The foundation for developing medications for the clinical therapy of COVID-19 is shown in Figure 1.

## 3. Results

### 3.1. Active Components and Target Proteins of Yinqiaosan

By a systematic search of the public databases, 141 components were retrieved in JYH (17), LQ (11), JG (7), BH (10), DDC (2), DZY (4), NBZ (4), JG (9), LG (1), and GC (76), and the specific information of these components can be found in Table 1. After deleting the duplicate values, 117 active ingredients were acquired. Refer to the results in Figure S1. Through observation, it was discovered that some special compounds in this network interacted with multiple targets and took part in the regulation of multiple targets, such as luteolin, naringenin, acacetin, kaempferol, and quercetin.

A total of 810 targets were obtained from public databases, but not all information on all 810 targets is shown. The YQS compounds may jointly affect all of these targets, producing pharmacological impacts on COVID-19.

### 3.2. Screening Results of COVID-19-Related Disease Targets

The GeneCards database, as an online catalog of human genes and genetic illness, can enable effective navigation of gene-disease linkages [23]. The OMIM database deals with over 15,500 gene entries and concentrates on elaborating gene-phenotype correlations [24]. Through the two databases, we obtained 435 genes in total (not all shown).

### 3.3. Screening Results of Candidate Targets

**Figure 2:**
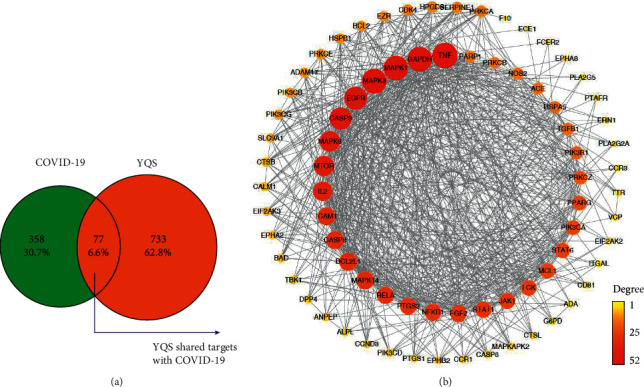
Overlapping targets (a) and the protein-protein network (b). Node colors change gradually from red to yellow, and node sizes are in proportion to their degree.

Altogether, 77 overlapping genes were acquired by searching the overlaps of the aforementioned compound targets with the 435 COVID-19 gene targets. These genes included PIK3CD, PLA2G5, and ECE1 (Table S1, Figure 2(a)).

By importing compound-disease cotarget information into STRING, a compound-COVID-19 target PPI network was obtained. Taking a score ≥0.4 as the limiting condition [19], the interaction relationship of 77 targets obtained from the String network database was screened. Then, a network diagram of 77 targets protein interactions was obtained. Combined with Cytoscape 3.5.1 software, we obtained an interaction network graph of alternative targets of YQS for treating COVID-19, including 77 nodes and 719 edges. The node colors are illustrated from red to yellow in descending order of degree values (Figure 2(b)). Among the targets of YQS for treating COVID-19, the 5 targets with the highest degree were TNF, GAPDH, MAPK1, MAPK3, and EGFR.

### 3.4. Results of the Construction of the Compound-Target Network

**Figure 3:**
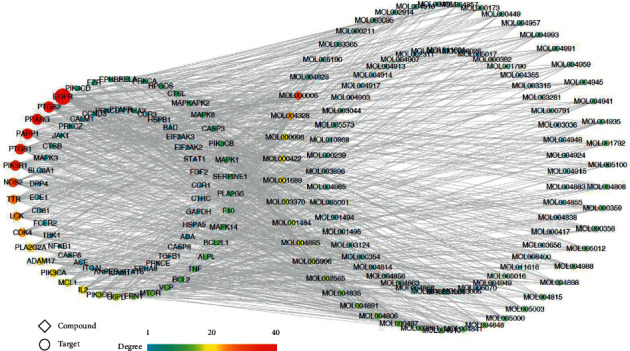
The compound-target networks of Yinqiaosan (YQS). The diamond nodes denote ingredients, and the circular nodes denote targets. The node colors from red to blue and node size are proportional to its degree.

The data of the active ingredients of YQS as well as its targets for treating COVID-19 were input into Cytoscape 3.5.1 software to obtain the network chart of active ingredient-acting targets. The active ingredients of YQS were represented by their Mol IDs. There were 173 nodes and 831 edges forming the compound-target network. The diamonds represent 95 active ingredients, and the ellipse indicates 77 targets of active ingredients (Figure 3).

### 3.5. Enrichment Analysis Results of Biological Processes and Pathways

**Figure 4:**
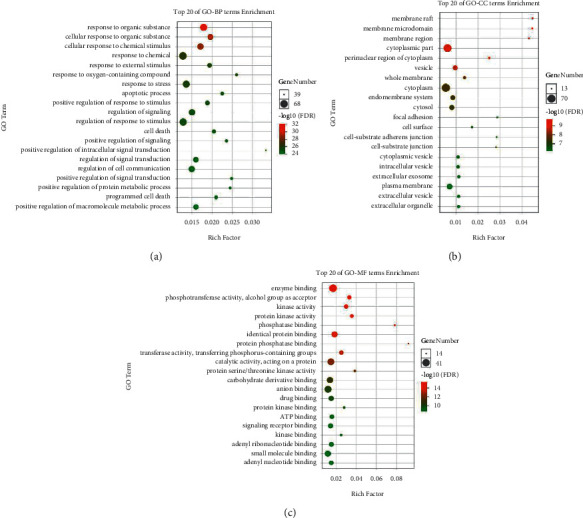
GO enrichment analysis of Yinqiaosan (YQS) targets. The three strata were biological processes (BP) (a), cellular components (CC) (b), and molecular functions (MF) (c). *Y*-axis: top 20 biological processes associated with the enriched targets. *X*-axis: enrichment factors (rich factors). Rich factors are proportional to the degree of enrichment. The node size shows the number of genes, and the larger the node is, the more the genes enriched in the term are. The color depth of the node demonstrates the significance level, and the redder the node is, the higher the significance will be to the term.

To carry out an in-depth recognition of the biological functions of the above 77 potential targets in biological networks, a GO enrichment analysis was implemented with the OmicShare tools, a free online platform for data analysis (http://www.omicshare.com/tools) [25]. As shown in Figure 4, the results fell into three strata: biological processes (BP), cellular components (CC), and molecular functions (MF). The limiting condition was *P* < 0.01, where the smaller the *P* value is, the closer the relationship with YQS will be in treating pneumonia. The results show that the BP analysis mainly includes the response to organic substances, the cellular response to organic substances, and the cellular response to chemical stimulus (Figure 4(a)). The CC analysis mainly included membrane microdomains, membrane rafts, and membrane regions (Figure 4(b)). The MF analysis was mainly related to enzyme binding, phosphotransferase activities, alcohol groups as acceptors, and kinase activities (Figure 4(c)).

**Figure 5:**
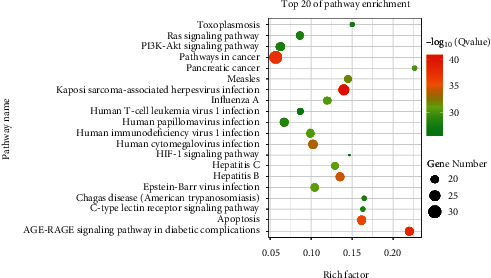
Analysis of the 20 most enriched KEGG pathways of candidate targets of Yinqiaosan (YQS) for COVID-19. The node size denotes the number of target genes that belong to the pathway. The *X*-axis denotes the enrichment factor (rich factor), that is, the number of genes in the pathway in the target gene/the number of genes contained in the pathway in the background gene set. The *Y*-axis denotes the pathway. The color depth of the node demonstrates the significance level, and the redder the node is, the higher the significance will be to the pathway.

Utilizing the online platform KOBAS 3.0, 190 important KEGG pathways (*P* < 0.01) were found on the basis of differentially expressed coding transcripts. The most important pathways included Kaposi sarcoma-related herpesvirus infection, AGE-RAGE signaling pathways in diabetic complications, and pathways in cancer. The result was constructed by KOBAS 3.0, and the top 20 enrichment pathways were visualized by RStudio (Figure 5). This indicated that YQS might be a candidate for treating COVID-19 through these pathways.

### 3.6. Construction of “YQS-Compounds-Targets-Pathway” Network

**Figure 6:**
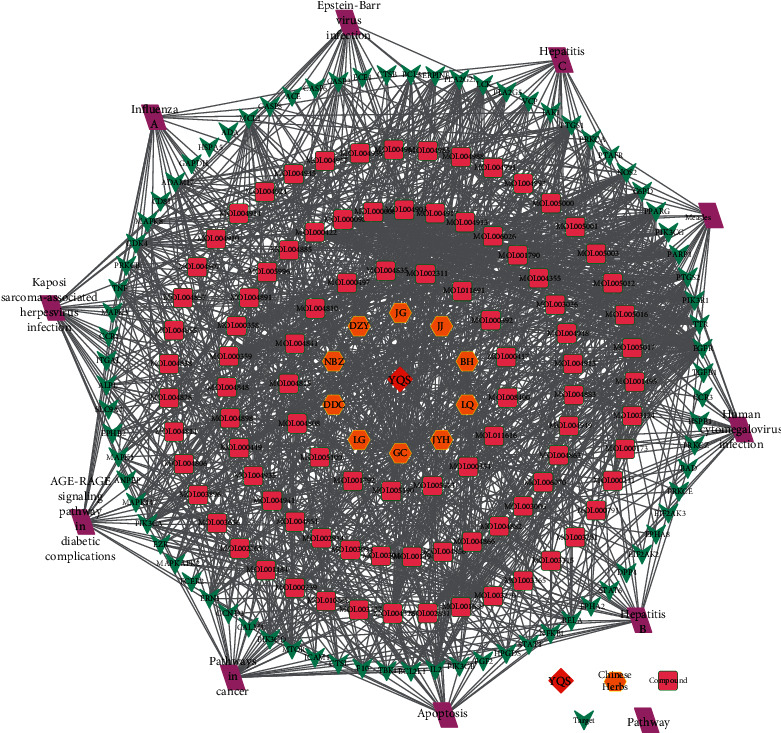
Yinqiaosan (YQS)-compound-target-pathway network. The red bubbles denote YQS (Yinqiaosan), the yellow bubbles denote Chinese herbs, the pink bubbles denote compounds, and the violet bubbles denote pathways.

The top ten channels in the enrichment analysis of the KEGG pathway and the compound and compound targets were imported into Cytoscape 3.5.1 software to build a “YQS-compounds-targets-pathway” network (Figure 6). The figure includes 194 nodes and 1185 edges. The red diamonds denote YQS, yellow hexagons represent the traditional Chinese herbs in YQS, green rectangles represent the potential active ingredients, blue ellipses represent YQS candidate treatment targets for COVID-19, and purple triangles represent the possible signaling pathways for the active ingredients to function. It can be seen from the figure that YQS includes 10 herbs and 95 active ingredients. It mainly acts on TNF, GAPDH, MAPK3, MAPK1, EGFR, CASP3, MAPK8, MTOR, IL-2, and MAPK14 and uses Kaposi sarcoma-related herpesvirus infection, AGE-RAGE signaling pathways in diabetic complications, pathways in cancer, apoptosis, hepatitis B, human cytomegalovirus signaling pathways such as infection, measles, and hepatitis C to treat pneumonia.

### 3.7. Molecular Docking

Molecular docking was further utilized in our research to estimate the interaction between components and targets to reduce the complexity and increase the precision of the constituent target network [26]. The cytoHubba control panel is a useful tool to retrieve subnetworks from the whole large PPI set [27]. Hub gene recognition was processed by the cytoHubba plug-in in Cytoscape software. All active ingredients in the top 5 online pharmacology degrees of freedom were taken as the research object. To determine the valid connecting effects between YQS-main active ingredients and their predicted targets, molecular docking was assessed using the YQS-hub genes connecting the energy. Molecular docking analyzed 10 hub genes (CASP3, EGFR, GAPDH, IL-2, MAPK1, MAPK14, MAPK3, MAPK8, mTOR, and TNF) and 5 compounds (acacetin, kaempferol, luteolin, naringenin, and quercetin).

Zhou et al. [3] ascertained that 2019-nCoV makes use of the same cell entry receptor, angiotensin transforming enzyme II (ACE2), as SARS-CoV. 3CL hydrolase is the core protease of single-stranded RNA virus precursor polyproteolysis and it plays a vital role when replicating the single-stranded RNA virus [28]. Therefore, in this study, the 5 compounds were also molecularly docked with SARS-CoV-2 3CL hydrolase (Mpro) and ACE2 to provide references for treating COVID-19 by TCM.

The lower the binding energy, the more stable the conformation of the ligand-receptor binding [29]. When the binding energy is less than −5.0 kcal/mol, this suggests that the compound can bind to the target. When the binding energy is less than -7.0 kcal/mol, this suggests that the compound has a good ability to bind to the target. We selected the conformation with the lowest binding energy to analyze the docking binding mode and used Discovery Studio for mapping.

**Figure 7:**
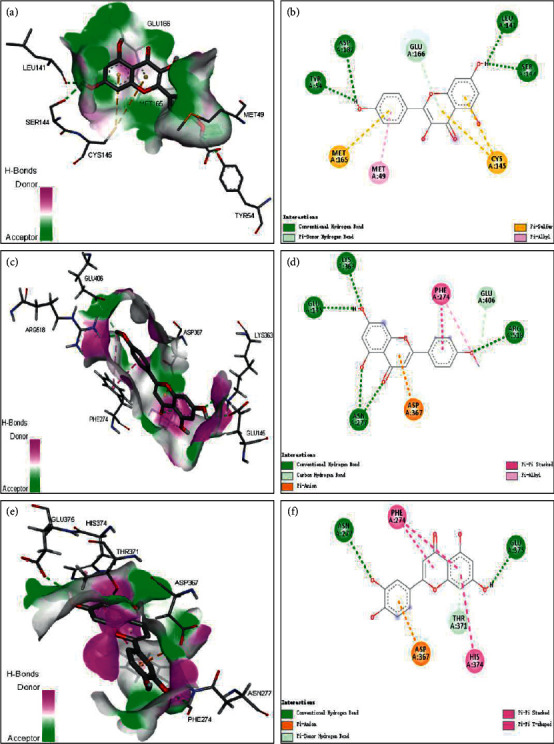
Molecular docking of compounds from YQS for COVID-19 targets. Predicted binding mode of Mpro (PDB id: 6lu7) with kaempferol in the active pocket (a). 2D binding view of kaempferol with Mpro (b). Predicted binding mode of ACE2 (PDB id: 1r4l) with acacetin in the active pocket (c). 2D binding view of acacetin with ACE2 (d). Predicted binding mode of ACE2 (PDB id: 1r4l) with luteolin in the active pocket (e). 2D binding view of luteolin with ACE2 (f).

Molecular docking results showed that all five active ingredients could be combined with 10 hub genes (Table S2), Mpro or ACE2 (Table 2, Figure 7). Therefore, the 5 compounds could efficiently act on the 10 targets, especially TNF, MAPK14, MAPK3, and MAPK8. Kaempferol (Figures 7(a) and 7(b)) and Mpro have a better binding capacity, which is consistent with the docking results of the molecules discovered by Ling [30]. Acacetin (Figures 7(c) and 7(d)), luteolin (Figures 7(e) and 7(f)), and ACE2 have a better combining ability.

## 4. Discussion

At present, COVID-19 has expanded to over 200 countries and areas, and the lives and health of people around the world are under serious threat. Special drugs against COVID-19 are still under development. TCM adopts the principle of dialectical treatment with the characteristics of multiple targets and multiple pathways, and it plays a vital role in antiepidemic treatment [31–33]. The “COVID-19 Diagnosis and Treatment Program (Trial Ver. 3)” issued by the National Health Commission of the People's Republic of China pointed out that COVID-19 is categorized as a “pestile” in TCM, which happens under direct or indirect contact with epidemic pathogens. The main features are “damp, heat, poisonous, stasis.” COVID-19 is divided into four types of TCM syndromes: cold-dampness stagnating in the lung, damp-heat accumulating in the lung, damp toxin stagnating in the lung, and internal blocking causing external collapse. The clinical symptoms of “damp-heat accumulating in the lung” are low body temperature or no fever, mild aversion to cold, fatigue, a heavy sensation in the head and body, dry coughs with scanty phlegm, sore throat, and a dry mouth with no wish to drink water. Alternatively, chest tightness, epigastric fullness, absence of sweating or inhibited sweating, vomiting, a poor appetite, and loose stools or sticky stools with a feeling of incomplete bowel movement may also be present. The tongue is pale red with a white, thick, and greasy or thin, yellow coating. The pulse is soft or slippery and rapid. The recommended prescriptions are Maxing shigantang and YQS.

YQS was described by Wu in “Wen Bing Tiao Bian.” He is a famous infectious febrile disease specialist. Part of the composition of the Lianhua Qingwen capsule comes from YQS, which is particularly effective in treating pneumonia. YQS is comprised of ten Chinese herbs, JYH, LQ, JG, BH, DDC, DZY, NBZ, JJ, LG, and GC, and mainly treats the manifestations caused by diseases such as damp-heat accumulation in the lung. Bohe and Niubangzi have flavor and cool properties and can help sore throats. Jingjie and Dandouchi can help the main medicine drive away damp heat. Dan Zhuye and Lugen can clear the heat, and Jiegeng and Gancao can relieve cough. YQS is commonly used in the clinic against influenza A virus infection [34–36].

This study uses network pharmacology to forecast the main active components and potential molecular mechanism of YQS against COVID-19. Through the TCMSP database, 141 active ingredients of each single medicine in Yinqiao powder were obtained, of which the five most powerful components were acacetin, kaempferol, luteolin, naringenin, and quercetin. Studies have shown that luteolin may suppress the infection of respiratory epithelial cells by *Escherichia coli* through immunomodulation [37]. Wu et al. established a mouse model of *Staphylococcus aureus* pneumonia and found that naringenin could significantly alleviate the disease [38]. Acacia has antibacterial, antioxidant, anti-inflammatory, antiviral, antipyretic, and analgesic effects [39]. It can protect against atherosclerosis by antagonizing the LysoPC-induced apoptosis of vascular smooth muscle cells and stimulating the antioxidant regulatory factor Nrf2 [40]. Kaempferol has an extensive scope of pharmacological activities, including antioxidant, anti-inflammatory, antimicrobial, etc. [41], and its derivatives are used as antiviral drugs against coronavirus 3a channel protein [42]. Quercetin and its derivatives also have strong anti-inflammatory and analgesic pharmacological activities [43]. Wang et al. [12] found that active compounds such as quercetin, kaempferol, naringenin, and luteolin also played a pivotal role when treating COVID-19 using Maxing Shigan decoction. Kong et al. [44] discovered through molecular docking that kaempferol, quercetin, luteolin, and new coronavirus (SARS-CoV-2) 3CL hydrolase have good affinity.

There are 77 candidate targets for YQS in COVID-19 treatment. TNF, GAPDH, MAPK3, MAPK1, EGFR, CASP3, MAPK8, mTOR, IL-2, and MAPK14 are important targets for YQS in COVID-19 treatment. COVID-19 is similar to other viral pneumonia conditions. Some scholars have found that cytokine storms (CSs) may affect the progression of COVID-19 illness [45]. The cytokines involved in CS mainly include six categories: tumor necrosis factor (TNF), interleukin (IL), interferon (IFN), colony stimulating factor (CSF), and growth factor (GF). The cytokine storm induced by SARS-CoV-2 includes the cytokine IL-2, and the TNF-*α*- and H7N9-induced cytokine storms include cytokines, including IL-2 [46]. Studies have shown that, in throat swabs and sputum specimens of 4 cases of COVID-19, the housekeeping gene GAPDH of human cells showed a typical amplification signal curve [47]. *Staphylococcus aureus* pneumonia and virulence factor A can stimulate the AMPK signaling pathway and inhibit the mTOR pathway to induce autophagy in alveolar epithelial cells, an important mechanism during early infection of *S. pneumoniae* [48]. Liu et al. [49] predicted MAPK1, MAPK3, and MAPK14 through network pharmacology as important targets for Dayuanyin to treat COVID-19. Huang et al. [4] predicted that MAPK1 and MAPK14 were important targets for Huanglian Jiedu decoction to treat COVID-19. Other studies have shown that EGFR may be a key factor in the inflammation caused by *Klebsiella pneumoniae* infection [50].

Furthermore, molecular docking results showed that the 5 compounds could efficiently act on the 10 targets, especially TNF, MAPK14, MAPK3, and MAPK8. Kaempferol and Mpro have a better binding capacity. Acacetin, luteolin, and ACE2 have a better combining ability. Whether they are docked with molecules of hub genes or docked with the proven targets of pneumonia, this shows the feasibility of using these five compounds to treat pneumonia. Reportedly, AGEs modulate COVID-19 pathogenesis and related comorbidities through their interactions with RAGE, among other molecules [51]. Interestingly, Yalcin Kehribar et al. [52] found that the RAGE pathway plays an important role in the aggravation of COVID-19. These reports provide strong evidence for our data.

To clarify the role of YQS in COVID-19 treatment based on gene functions and signaling pathways, this study performed GO functional enrichment and pathway enrichment analysis of the candidate targets. The GO enrichment analysis showed that the biological procedure of YQS against COVID-19 was mainly reflected in the positive regulation of the adhesion of white blood cells and vascular endothelial cells and the negative regulation of the apoptosis process of cholangiocarcinoma cells. The cellular components were mainly reflected in cytoplasmic vesicles, membrane rafts, and organelle membranes. The molecular functions were mainly reflected in phosphotransferase activity, alcohol receptor, and cytokine receptor binding. The results of KEGG pathway enrichment analysis show that YQS may comprehensively play a role in preventing and treating pneumonia by way of PTGS2, MAPK14, CCR1, CCR3, MAPKAPK2, MAPK3, MAPK1, ICAM1, JAK1, NFKB1, MAPK8, PIK3R1, MTOR, RELA, CASP3, FGF2, TBK1, EIF2AK2, CASP8, and CDK4 targets that are related to the Kaposi sarcoma-associated herpesvirus infection signaling pathway; TGFB1, MAPK14, SERPINE1, STAT1, PIK3CA, PIK3CB, PIK3CD, MAPK3, MAPK1, PIK3R1, NFKB1, RELA, ICAM1, PRKCA, PRKCB, PRKCE, PRKCZ, MAPK8, CASP3, TNF, CDK4, and BCL2 targets that are related to the AGE-RAGE signaling pathway; and TGFB1, MAPK14, SERPINE1, STAT1, PIK3CA, PIK3CB, PIK3CD, MAPK3, MAPK1, PIK3R1, NFKB1, RELA, ICAM1, PRKCA, PRKCB, PRKCE, PRKCZ, MAPK8, CASP3, TNF, CDK4, and BCL2 targets that are related to the pathways in cancer signaling pathways.

## 5. Summary

In summary, our study found that active ingredients such as luteolin, naringenin, farnesin, kaempferol, and quercetin can act on targets such as TNF, GAPDH, MAPK3, MAPK1, EGFR, CASP3, MAPK8, MTOR, IL-2, and MAPK14 to regulate signaling pathways such as Kaposi sarcoma-associated herpesvirus infection and AGE-RAGE, finally achieving the effect of curing COVID-19.

In the next step, further in vitro and in vivo experimental validation will be carried out to advance our research. The follow-up plan of our research group is to carry out more in-depth experimental research on YQS and to fully explore the mechanism of its efficacy.

## Figures and Tables

**Table 1:** Active ingredients of Yinqiaosan (YQS).

Chinese herbs	Mol id	Molecule name	OB (%)	DL	HL(h)
JYH	MOL000006	Luteolin	36.16	0.25	15.94
JYH	MOL000098	Quercetin	46.43	0.28	14.4
JYH	MOL000358	Beta-sitosterol	36.91	0.75	5.36
JYH	MOL000422	Kaempferol	41.88	0.24	14.74
JYH	MOL000449	Stigmasterol	43.83	0.76	5.57
JYH	MOL001494	Mandenol	42	0.19	5.39
JYH	MOL001495	Ethyl linolenate	46.1	0.2	6.2
JYH	MOL002773	Beta-carotene	37.18	0.58	4.36
JYH	MOL002914	Eriodyctiol (flavanone)	41.35	0.24	15.88
JYH	MOL003006	(-)-(3R,8S,9R,9aS,10aS)-9-Ethenyl-8-(beta-D-glucopyranosyloxy)-2,3,9,9a,10,10a-hexahydro-5-oxo-5H,8H-pyrano[4,3-d]oxazolo[3,2-a]pyridine-3-carboxylic acid_qt	87.47	0.23	5.5
JYH	MOL003036	ZINC03978781	43.83	0.76	5.79
JYH	MOL003044	Chrysoeriol	35.85	0.27	16.31
JYH	MOL003059	kryptoxanthin	47.25	0.57	4.37
JYH	MOL003062	4,5′-Retro-.beta.,.beta.-Carotene-3,3′-dione, 4′,5′-didehydro-	31.22	0.55	5.39
JYH	MOL003095	5-hydroxy-7-methoxy-2-(3,4,5-trimethoxyphenyl)chromone	51.96	0.41	15.98
JYH	MOL003111	Centauroside_qt	55.79	0.5	5.18
JYH	MOL003124	XYLOSTOSIDINE	43.17	0.64	9.15
LQ	MOL000006	luteolin	36.16	0.25	15.94
LQ	MOL000098	quercetin	46.43	0.28	14.4
LQ	MOL000173	wogonin	30.68	0.23	17.75
LQ	MOL000211	Mairin	55.38	0.78	8.87
LQ	MOL000358	Beta-sitosterol	36.91	0.75	5.36
LQ	MOL000422	Kaempferol	41.88	0.24	14.74
LQ	MOL000791	Bicuculline	69.67	0.88	15.83
LQ	MOL003281	20(S)-Dammar-24-ene-3*β*,20-diol-3-acetate	40.23	0.82	9.14
LQ	MOL003315	3beta-Acetyl-20,25-epoxydammarane-24alpha-ol	33.07	0.79	7.82
LQ	MOL003365	Lactucasterol	40.99	0.85	5.53
LQ	MOL003370	Onjixanthone I	79.16	0.3	14.86
JG	MOL000006	Luteolin	36.16	0.25	15.94
JG	MOL001689	Acacetin	34.97	0.24	17.25
JG	MOL004355	Spinasterol	42.98	0.76	5.32
JG	MOL004580	cis-Dihydroquercetin	66.44	0.27	14.51
JG	MOL005996	2-O-Methyl-3―O-*β*-D-glucopyranosyl platycogenate A	45.15	0.25	6.03
JG	MOL006026	Dimethyl 2-O-methyl-3-O-a-D-glucopyranosyl platycogenate A	39.21	0.25	5.04
JG	MOL006070	Robinin	39.84	0.71	16.67
BH	MOL000006	Luteolin	36.16	0.25	15.94
BH	MOL000359	Sitosterol	36.91	0.75	5.37
BH	MOL000471	Aloe-emodin	83.38	0.24	31.49
BH	MOL001689	Acacetin	34.97	0.24	17.25
BH	MOL001790	Linarin	39.84	0.71	16.07
BH	MOL002881	Diosmetin	31.14	0.27	16.34
BH	MOL004328	Naringenin	59.29	0.21	16.98
BH	MOL005190	Eriodictyol	71.79	0.24	15.81
BH	MOL005573	Genkwanin	37.13	0.24	16.1
BH	MOL011616	Fortunellin	35.65	0.74	14.19
DDC	MOL008400	Glycitein	50.48	0.24	16.32
DDC	MOL011691	6′-O-Malonylglycitin	30.4	0.81	17.25
DZY	MOL000006	Luteolin	36.16	0.25	15.94
DZY	MOL000359	Sitosterol	36.91	0.75	5.37
DZY	MOL002322	Isovitexin	31.29	0.72	16.45
DZY	MOL003137	Swertiajaponin	32.12	0.78	16.28
NBZ	MOL000358	Beta-sitosterol	36.91	0.75	5.36
NBZ	MOL000422	Kaempferol	41.88	0.24	14.74
NBZ	MOL002773	Beta-carotene	37.18	0.58	4.36
NBZ	MOL010868	Neoarctin A	39.99	0.27	5.82
JJ	MOL000006	Luteolin	36.16	0.25	15.94
JJ	MOL000098	Quercetin	46.43	0.28	14.4
JJ	MOL000358	Beta-sitosterol	36.91	0.75	5.36
JJ	MOL000359	Sitosterol	36.91	0.75	5.37
JJ	MOL000449	Stigmasterol	43.83	0.76	5.57
JJ	MOL002881	Diosmetin	31.14	0.27	16.34
JJ	MOL005043	Campest-5-en-3beta-ol	37.58	0.71	4.43
JJ	MOL005100	5,7-Dihydroxy-2-(3-hydroxy-4-methoxyphenyl)chroman-4-one	47.74	0.27	16.51
JJ	MOL011856	Schkuhrin I	54.45	0.52	5.89
LG	MOL000449	Stigmasterol	43.83	0.76	5.57
GC	MOL000098	Quercetin	46.43	0.28	14.4
GC	MOL000211	Mairin	55.38	0.78	8.87
GC	MOL000239	Jaranol	50.83	0.29	15.5
GC	MOL000354	Isorhamnetin	49.6	0.31	14.34
GC	MOL000359	Sitosterol	36.91	0.75	5.37
GC	MOL000392	Formononetin	69.67	0.21	17.04
GC	MOL000417	Calycosin	47.75	0.24	17.1
GC	MOL000422	Kaempferol	41.88	0.24	14.74
GC	MOL000497	Licochalcone a	40.79	0.29	16.2
GC	MOL001484	Inermine	75.18	0.54	11.72
GC	MOL001792	DFV	32.76	0.18	17.89
GC	MOL002311	Glycyrol	90.78	0.67	9.85
GC	MOL002565	Medicarpin	49.22	0.34	8.46
GC	MOL003656	Lupiwighteone	51.64	0.37	15.63
GC	MOL003896	7-Methoxy-2-methyl isoflavone	42.56	0.2	16.89
GC	MOL004328	Naringenin	59.29	0.21	16.98
GC	MOL004805	(2S)-2-[4-Hydroxy-3-(3-methylbut-2-enyl)phenyl]-8,8-dimethyl-2,3-dihydropyrano[2,3-f]chromen-4-one	31.79	0.72	14.82
GC	MOL004806	Euchrenone	30.29	0.57	15.89
GC	MOL004808	Glyasperin B	65.22	0.44	16.1
GC	MOL004810	Glyasperin F	75.84	0.54	15.64
GC	MOL004814	Isotrifoliol	31.94	0.42	7.91
GC	MOL004815	(E)-1-(2,4-Dihydroxyphenyl)-3-(2,2-dimethylchromen-6-yl)prop-2-en-1-one	39.62	0.35	16.16
GC	MOL004824	(2S)-6-(2,4-dihydroxyphenyl)-2-(2-hydroxypropan-2-yl)-4-methoxy-2,3-dihydrofuro[3,2-g]chromen-7-one	60.25	0.63	4.31
GC	MOL004827	Semilicoisoflavone B	48.78	0.55	17.02
GC	MOL004828	Glepidotin A	44.72	0.35	16.09
GC	MOL004829	Glepidotin B	64.46	0.34	15.98
GC	MOL004835	Glypallichalcone	61.6	0.19	17.01
GC	MOL004838	8-(6-Hydroxy-2-benzofuranyl)-2,2-dimethyl-5-chromenol	58.44	0.38	8.71
GC	MOL004841	Licochalcone B	76.76	0.19	17.02
GC	MOL004848	Licochalcone G	49.25	0.32	15.75
GC	MOL004855	Licoricone	63.58	0.47	16.37
GC	MOL004856	Gancaonin A	51.08	0.4	16.82
GC	MOL004857	Gancaonin B	48.79	0.45	16.49
GC	MOL004860	Licorice glycoside E	32.89	0.27	25.39
GC	MOL004863	3-(3,4-Dihydroxyphenyl)-5,7-dihydroxy-8-(3-methylbut-2-enyl)chromone	66.37	0.41	15.81
GC	MOL004864	5,7-Dihydroxy-3-(4-methoxyphenyl)-8-(3-methylbut-2-enyl)chromone	30.49	0.41	14.99
GC	MOL004866	2-(3,4-Dihydroxyphenyl)-5,7-dihydroxy-6-(3-methylbut-2-enyl)chromone	44.15	0.41	16.77
GC	MOL004882	Licocoumarone	33.21	0.36	9.66
GC	MOL004883	Licoisoflavone	41.61	0.42	16.09
GC	MOL004884	Licoisoflavone B	38.93	0.55	15.73
GC	MOL004885	Licoisoflavanone	52.47	0.54	15.67
GC	MOL004891	Shinpterocarpin	80.3	0.73	6.5
GC	MOL004898	(E)-3-[3,4-Dihydroxy-5-(3-methylbut-2-enyl)phenyl]-1-(2,4-dihydroxyphenyl)prop-2-en-1-one	46.27	0.31	15.24
GC	MOL004903	Liquiritin	65.69	0.74	17.96
GC	MOL004907	Glyzaglabrin	61.07	0.35	21.2
GC	MOL004910	Glabranin	52.9	0.31	16.24
GC	MOL004912	Glabrone	52.51	0.5	16.09
GC	MOL004913	1,3-Dihydroxy-9-methoxy-6-benzofurano[3,2-c]chromenone	48.14	0.43	8.87
GC	MOL004914	1,3-Dihydroxy-8,9-dimethoxy-6-benzofurano[3,2-c]chromenone	62.9	0.53	9.32
GC	MOL004915	Eurycarpin A	43.28	0.37	17.1
GC	MOL004917	Glycyroside	37.25	0.79	14.62
GC	MOL004924	(-)-Medicocarpin	40.99	0.95	13.2
GC	MOL004935	Sigmoidin-B	34.88	0.41	14.49
GC	MOL004941	(2R)-7-Hydroxy-2-(4-hydroxyphenyl)chroman-4-one	71.12	0.18	18.09
GC	MOL004945	(2S)-7-Hydroxy-2-(4-hydroxyphenyl)-8-(3-methylbut-2-enyl)chroman-4-one	36.57	0.32	17.95
GC	MOL004948	Isoglycyrol	44.7	0.84	6.69
GC	MOL004949	Isolicoflavonol	45.17	0.42	15.55
GC	MOL004957	HMO	38.37	0.21	16.56
GC	MOL004959	1-Methoxyphaseollidin	69.98	0.64	9.53
GC	MOL004961	Quercetin der.	46.45	0.33	16.61
GC	MOL004985	Icos-5-enoic acid	30.7	0.2	5.28
GC	MOL004988	Kanzonol F	32.47	0.89	9.98
GC	MOL004989	6-Prenylated eriodictyol	39.22	0.41	16.52
GC	MOL004991	7-Acetoxy-2-methylisoflavone	38.92	0.26	17.49
GC	MOL004993	8-Prenylated eriodictyol	53.79	0.4	15.7
GC	MOL004996	Gadelaidic acid	30.7	0.2	5.25
GC	MOL005000	Gancaonin G	60.44	0.39	16.13
GC	MOL005001	Gancaonin H	50.1	0.78	16.64
GC	MOL005003	Licoagrocarpin	58.81	0.58	9.45
GC	MOL005007	Glyasperins M	72.67	0.59	15.57
GC	MOL005008	Glycyrrhiza flavonol A	41.28	0.6	13.71
GC	MOL005012	Licoagroisoflavone	57.28	0.49	19.64
GC	MOL005013	18*α*-Hydroxyglycyrrhetic acid	41.16	0.71	4.96
GC	MOL005016	Odoratin	49.95	0.3	16.35
GC	MOL005017	Phaseol	78.77	0.58	9.64
GC	MOL005018	Xambioona	54.85	0.87	14.5

**Table 2:** Docking results of main ingredients with target proteins.

No.	Ligand name	Mol id	Binding free energy/(kcal·mol^−1^)	
			Mpro	ACE2
1	Acacetin	MOL001689	−7.5	−8.8
2	Kaempferol	MOL000422	−7.9	−8.6
3	Luteolin	MOL000006	−7.4	−8.8
4	Naringenin	MOL004328	−6.8	−7.9
5	Quercetin	MOL000098	−7.5	−8.5

## Data Availability

All data included in this study are available upon request by contact with the corresponding author.

## Supplementary Materials

Supplementary Materials Supplementary 1. Additional file 1: Fig. S1: YQS (Yinqiaosan) formula-active ingredient network diagram. Supplementary 2. Additional file 2: Table S1: the information of the related targets of YQS (Yinqiaosan). Supplementary 3. Additional file 3: Table S2: virtual docking of five bioactive ingredients from YQS for COVID-19 targets.
