# Supplementary material for: Using a System Pharmacology Method to Search for the Potential Targets and Pathways of Yinqiaosan against COVID-19
Source: J Healthc Eng. 2022 Mar 15;2022:9248674. doi: 10.1155/2022/9248674 (PMC8941516; doi:10.1155/2022/9248674)
Supplement: Supplementary Materials — Supplementary 1. Additional file 1: Fig. S1: YQS (Yinqiaosan) formula-active ingredient network diagram. Supplementary 2. Additional file 2: Table S1: the information of the related targets of YQS (Yinqiaosan). Supplementary 3. Additional file 3: Table S2: virtual docking of five bioactive ingredients from YQS for COVID-19 targets. [file 9248674.f1.zip › 9248674.f1/Additional file 1.docx]

**Supplementary materials**


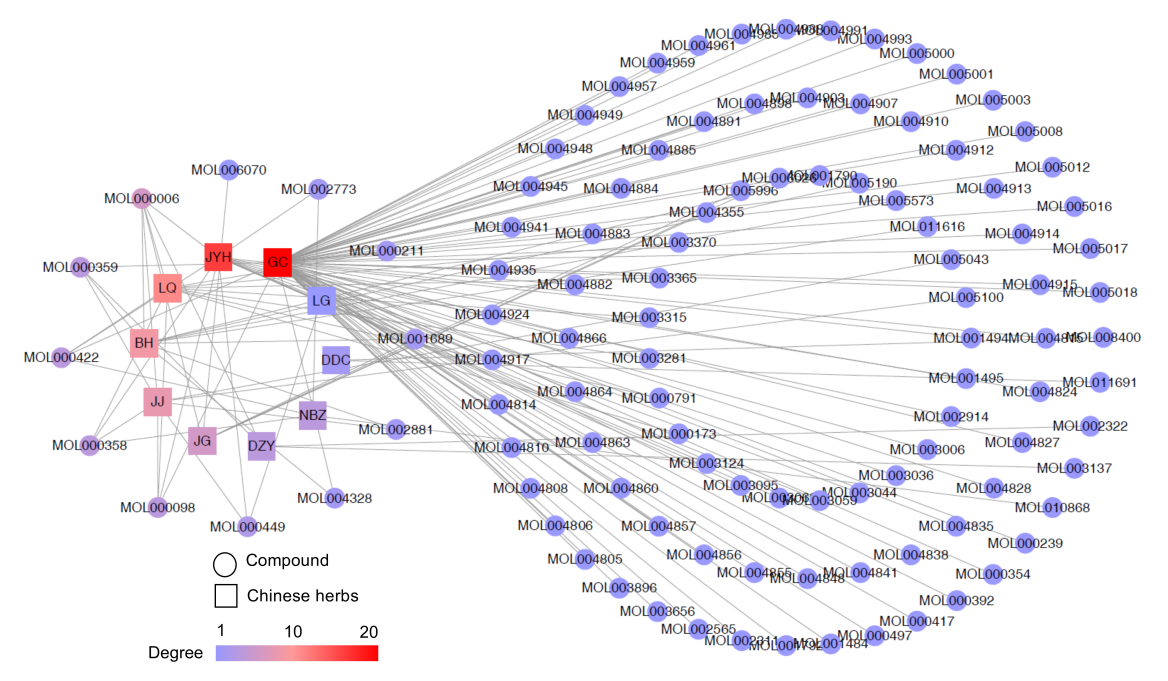


Fig. S1 YQS (Yinqiaosan) formula-active ingredient network diagram. The triangles represent herbs and the squares represent compounds. The colors of the nodes are illustrated from red to purple in descending order of degree values.
