# Supplementary material for: Using a System Pharmacology Method to Search for the Potential Targets and Pathways of Yinqiaosan against COVID-19
Source: J Healthc Eng. 2022 Mar 15;2022:9248674. doi: 10.1155/2022/9248674 (PMC8941516; doi:10.1155/2022/9248674)
Supplement: Supplementary Materials — Supplementary 1. Additional file 1: Fig. S1: YQS (Yinqiaosan) formula-active ingredient network diagram. Supplementary 2. Additional file 2: Table S1: the information of the related targets of YQS (Yinqiaosan). Supplementary 3. Additional file 3: Table S2: virtual docking of five bioactive ingredients from YQS for COVID-19 targets. [file 9248674.f1.zip › 9248674.f1/Additional file 2.docx]

**Supplementary materials**

**Table S1 The infromation of the related targents of Yinqiansan**

| **Gene** | **Protein name** | **Uniprot ID** | **Gene** | **Protein name** | **Uniprot ID** |
| --- | --- | --- | --- | --- | --- |
| PIK3CD | Phosphatidylinositol 4,5-bisphosphate 3-kinase catalytic subunit delta isoform | O00329 | PTGS2 | Prostaglandin G/H synthase 2 | P35354 |
| PLA2G5 | Calcium-dependent phospholipase A2 | P39877 | BCL2 | Apoptosis regulator Bcl-2 | P10415 |
| ECE1 | Endothelin-converting enzyme 1 | P42892 | HSPA5 | Endoplasmic reticulum chaperone BiP | P11021 |
| ALPL | Alkaline phosphatase, tissue-nonspecific isozyme | P05186 | CDK4 | Cyclin-dependent kinase 4 | P11802 |
| PTAFR | Platelet-activating factor receptor | P25105 | G6PD | Glucose-6-phosphate 1-dehydrogenase | P11413 |
| LCK | Tyrosine-protein kinase Lck | P06239 | PARP1 | Poly [ADP-ribose] polymerase 1 | P09874 |
| PRKCZ | Protein kinase C zeta type | Q05513 | RELA | Transcription factor p65 | Q04206 |
| MTOR | Serine/threonine-protein kinase mTOR | P42345 | NOS2 | Nitric oxide synthase, inducible | P35228 |
| EPHA2 | Ephrin type-A receptor 2 | P29317 | EIF2AK2 | Interferon-induced, double-stranded RNA-activated protein kinase | P19525 |
| SLC9A1 | Sodium/hydrogen exchanger 1 | P19634 | GAPDH | Glyceraldehyde-3-phosphate dehydrogenase | P04406 |
| PLA2G2A | Phospholipase A2, membrane associated | P14555 | CTSB | Cathepsin B | P07858 |
| EPHA8 | Ephrin type-A receptor 8 | P29322 | CASP8 | Caspase-8 | Q14790 |
| EPHB2 | Ephrin type-B receptor 2 | P29323 | BAD | Bcl2-associated agonist of cell death | Q92934 |
| TNF | Tumor necrosis factor | P01375 | ADAM17 | Disintegrin and metalloproteinase domain-containing protein 17 | P78536 |
| CASP3 | Caspase-3 | P42574 | HSPB1 | Heat shock protein beta-1 | P04792 |
| STAT1 | Signal transducer and activator of transcription 1-alpha/beta | P42224 | CCR1 | C-C chemokine receptor type 1 | P32246 |
| DPP4 | Dipeptidyl peptidase 4 | P27487 | EZR | Ezrin | P15311 |
| MCL1 | Induced myeloid leukemia cell differentiation protein Mcl-1 | Q07820 | VCP | Transitional endoplasmic reticulum ATPase | P55072 |
| CASP6 | Caspase-6 | P55212 | ITGAL | Integrin alpha-L | P20701 |
| IL2 | Interleukin-2 | P60568 | STAT6 | Signal transducer and activator of transcription 6 | P42226 |
| EGFR | Epidermal growth factor receptor | P00533 | PIK3CG | Phosphatidylinositol 4,5-bisphosphate 3-kinase catalytic subunit gamma isoform | P48736 |
| TGFB1 | Transforming growth factor beta-1 proprotein | P01137 | PRKCA | Protein kinase C alpha type | P17252 |
| PPARG | Peroxisome proliferator-activated receptor gamma | P37231 | CCR3 | C-C chemokine receptor type 3 | P51677 |
| MAPK1 | Mitogen-activated protein kinase 1 | P28482 | MAPKAPK2 | MAP kinase-activated protein kinase 2 | P49137 |
| FGF2 | Fibroblast growth factor 2 | P09038 | BCL2L1 | Bcl-2-like protein 1 | Q07817 |
| ACE | Angiotensin-converting enzyme | P12821 | CD81 | CD81 antigen | P60033 |
| ICAM1 | Intercellular adhesion molecule 1 | P05362 | PTGS1 | Prostaglandin G/H synthase 1 | P23219 |
| MAPK3 | Mitogen-activated protein kinase 3 | P27361 | F10 | Coagulation factor X | P00742 |
| MAPK8 | Mitogen-activated protein kinase 8 | P45983 | JAK1 | Tyrosine-protein kinase JAK1 | P23458 |
| TTR | Transthyretin | P02766 | TBK1 | Serine/threonine-protein kinase TBK1 | Q9UHD2 |
| CTSL | Cathepsin L1 | P07711 | FCER2 | Low affinity immunoglobulin epsilon Fc receptor | P06734 |
| ANPEP | Aminopeptidase N | P15144 | PIK3CB | Phosphatidylinositol 4,5-bisphosphate 3-kinase catalytic subunit beta isoform | P42338 |
| PIK3R1 | Phosphatidylinositol 3-kinase regulatory subunit alpha | P27986 | PRKCE | Protein kinase C epsilon type | Q02156 |
| ADA | Adenosine deaminase | P00813 | ERN1 | Serine/threonine-protein kinase/endoribonuclease IRE1 | O75460 |
| MAPK14 | Mitogen-activated protein kinase 14 | Q16539 | EIF2AK3 | Eukaryotic translation initiation factor 2-alpha kinase 3 | Q9NZJ5 |
| NFKB1 | Nuclear factor NF-kappa-B p105 subunit | P19838 | CCND3 | G1/S-specific cyclin-D3 | P30281 |
| SERPINE1 | Plasminogen activator inhibitor 1 | P05121 | PRKCB | Protein kinase C beta type | P05771 |
| PIK3CA | Phosphatidylinositol 4,5-bisphosphate 3-kinase catalytic subunit alpha isoform | P42336 | CALM1 | Calmodulin-1 | P0DP23 |
| HPGDS | Hematopoietic prostaglandin D synthase | O60760 |  |  |  |
