# Supplementary material for: Using a System Pharmacology Method to Search for the Potential Targets and Pathways of Yinqiaosan against COVID-19
Source: J Healthc Eng. 2022 Mar 15;2022:9248674. doi: 10.1155/2022/9248674 (PMC8941516; doi:10.1155/2022/9248674)
Supplement: Supplementary Materials — Supplementary 1. Additional file 1: Fig. S1: YQS (Yinqiaosan) formula-active ingredient network diagram. Supplementary 2. Additional file 2: Table S1: the information of the related targets of YQS (Yinqiaosan). Supplementary 3. Additional file 3: Table S2: virtual docking of five bioactive ingredients from YQS for COVID-19 targets. [file 9248674.f1.zip › 9248674.f1/Additional file 3.docx]

**Supplementary materials**

**Table S2 Virtual Docking of Five Bioactive Ingredients From YQS For COVID-19 Targets**

| **No.** | **Ligand name** | **MOL ID** | **Binding free energy/(kcal∙mol^−1^)** | | | | | | | | | |
| --- | --- | --- | --- | --- | --- | --- | --- | --- | --- | --- | --- | --- |
|  |  |  | CASP3 | EGFR | GAPDH | IL-2 | MAPK1 | MAPK14 | MAPK3 | MAPK8 | MTOR | TNF |
| 1 | acacetin | MOL001689 | -6.1 | -8.3 | -6.1 | -5.1 | -7.6 | -9.4 | -9 | -9.2 | -7.8 | -9.6 |
| 2 | kaempferol | MOL000422 | -6.4 | -8.6 | -6.6 | -4.9 | -7.9 | -9.3 | -9 | -8.8 | -7.2 | -10.1 |
| 3 | luteolin | MOL000006 | -6.1 | -8.9 | -6.7 | -5.1 | -7.9 | -9.1 | -9.3 | -9 | -7.9 | -9.9 |
| 4 | naringenin | MOL004328 | -6.5 | -7.3 | -6.4 | -5.3 | -6.7 | -7.5 | -7.7 | -7.2 | -6.5 | -9.8 |
| 5 | quercetin | MOL000098 | -6 | -8.9 | -6.5 | -4.8 | -8 | -8.6 | -9 | -8.8 | -7.3 | -10.1 |
